# Buyang Huanwu Decoction Ameliorates Bleomycin-Induced Pulmonary Fibrosis in Rats via Downregulation of Related Protein and Gene Expression

**DOI:** 10.1155/2018/9185485

**Published:** 2018-02-28

**Authors:** Xuan Wang, Xia Li, Li-na Wang, Jing-juan Pan, Xue Yang, Yang-lin Wei

**Affiliations:** ^1^Department of Traditional Chinese Medicine, Changhai Hospital of Shanghai, Second Military Medical University, Shanghai, China; ^2^Shanghai University of Traditional Chinese Medicine, Shanghai, China; ^3^Baoshan Hospital of Integrated Traditional Chinese and Western Medicine, Shanghai, China

## Abstract

Little is known about the effects of Buyang Huanwu decoction on pulmonary fibrosis. Herein, 144 healthy SD rats were randomly divided into six groups: blank control group (B), model control group (M), positive medicine control group (Mp), and high-, moderate-, and low-dose Buyang Huanwu decoction groups (Hd, Md, and Ld). A pulmonary fibrosis model was established by endotracheal injection of bleomycin. On the second day of modeling, the corresponding saline, methylprednisolone suspension, and the three doses of Buyang Huanwu decoction were used to treat the 6 groups of rats by intragastric administration for 7, 14, and 28 consecutive days. After 7, 14, and 28 days of treatment, the mRNA expression of CTGF and AKT, the protein level of CTGF, p-AKT, and collagen types I and III were tested. Finally, we found that the serum collagen type I and III level in Hd, Md, and Ld rats on the 14th and 28th day and the collagen type I and III level in Hd rats on 7th day were significantly lower than in M rats (*P* < 0.01). The protein level of p-AKT and CTGF in Hd and Md rats on the 7th and 14th days and the protein level of p-AKT in Hd rats on the 28th day were lower than in M rats (*P* < 0.01, *P* < 0.05). The level of CTGF mRNA in Hd, Md, and Ld rats and the level of AKT mRNA in Hd and Md rats on the 7th, 14th, and 28th days and the expression level of AKT mRNA in Ld rats on the 14th and 28th days were significantly lower than in M rats (*P* < 0.01). The study suggests that Buyang Huanwu decoction alleviated pulmonary fibrosis of rats by improvement of lung tissue morphology, low level of serum collagen types I and III, and the reduced expression of CTGF and p-AKT protein, which might be a result of its downregulated expression of CTGF and AKT mRNA levels.

## 1. Introduction

Pulmonary fibrosis (PF) is characterized by massive inflammatory cells infiltration, aberrant proliferation of fibroblasts, damage of alveolar structures, and excessive collagen accumulation [1]. New drugs (nintedanib and pirfenidone) have been recommended to treat pulmonary fibrosis. However, deleterious side effects of conventional and chemical drugs are motivating factors for many to seek herbal treatment for pulmonary fibrosis, which may be associated with fewer adverse effects [2]. The most common histopathological pattern seen in lung-biopsy specimens from patients with idiopathic pulmonary fibrosis is termed usual interstitial pneumonia and is characterized by heterogeneous areas of dense fibrosis, the presence of fibroblastic foci, and honeycombing with architectural distortion [3]. It is a relentlessly progressive and ultimately fatal disorder with a dismal median survival of 2 to 3 years from the time of diagnosis [3, 4]. The mechanisms in PF are unknown but it is speculated that persistent lung injury leads to alveolar epithelial cell injury and death, and subsequent aberrant repair mechanism(s) ablates the alveolus. In recent years, studies confirm that CTGF, as the downstream effect of TGF-*β*1 molecules, participated in the transformation process of lung fibroblasts to muscle fibroblasts and can be mediated by fibroblast proliferation and extracellular matrix deposition, which plays a key role in pulmonary fibrosis [5, 6].

A large amount of scientific research work contributed to the identification of whether PI3K/AKT signaling pathway plays a critical role in lung injury and repair. When normal fibroblasts attach to monomeric type I collagen, integrin receptors signal the downregulation of PTEN and activation of the PI3K/AKT protein phosphorylation cascade [7]. In contrast, interactions with polymerized collagen do not signal decreasing PTEN and the activity of PI3K/AKT remains low. The occurrence and development of PF are closely related to the expression of certain genes. This animal experiment focuses on the effect, protein level, and gene expression of Buyang Huanwu decoction in the treatment of pulmonary fibrosis.

## 2. Methods

### 2.1. Materials and Reagents

Buyang Huanwu decoction, which includes Radix Astragali, Radix Paeoniae Rubra, Rhizoma Ligustici Wallichii,* Angelica*, earthworm, peach kernel, and safflower, was purchased from Changhai Hospital of Shanghai and condensed into 1.0, 0.5, and 0.25 g/ml decoctions. Bleomycin was purchased from Nippon Kayaku Co. Ltd. (batch number: Y50512). Methylprednisolone was obtained from Pfizer Italia (batch number: Z829A).

### 2.2. Animals and Grouping

144 adult male SD rats (220 ± 20 g) were purchased from Shanghai Sippr-BK Laboratory Animal Co. Ltd. (certification number: SCXK Hu 20130016). All animal procedures used in the present study were reviewed and approved by the Institutional Animal Care and Use Committee. According to the random number table, all rats were divided into six groups randomly: blank control group (B), model control group (M), positive medicine (methylprednisolone) control group (Mp), and high-, moderate-, and low-dose Buyang Huanwu decoction groups (Hd, Md, and Ld). Each group included 24 rats. All of the rats were kept in a controlled room with 12 h light/dark cycle. The room temperature was kept at a constant 22–25°C temperature and 45–60% relative humidity. Rats were allowed free access to food and water. Except for the blank control group animals, all of the animals in the other five groups received 5 mg/kg 5% BLM by endotracheal injection to establish a pulmonary fibrosis model. 0.9% normal saline in the same volume was injected into the blank control group rats in the same way. Starting from the second day after modeling, rats in the blank and model control groups were treated with 1 ml/100 g 0.9% normal saline once daily, rats in the positive medicine control group were treated with 5 mg/kg methylprednisolone (0.9% normal saline suspension liquid: 0.5 mg/ml) once daily, and rats in the high-, moderate-, and low-dose Buyang Huanwu decoction groups were treated with 10, 5, and 2.5 g/kg Buyang Huanwu decoction once daily. All drugs were applied by intragastric administration. After 7, 14, and 28 days of drug treatment, 8 rats in each group were sacrificed randomly using intraperitoneal injection of 10% chloral hydrate (0.4 ml/100 g), and 5 ml of blood was sampled from the abdominal aorta and then centrifuged. Afterwards, serum samples were stored at −20°C. Meanwhile, a 1.5 cm × 1.5 cm × 0.3 cm middle lobe of the right and left lungs was, respectively, sampled, washed clean, cut into pieces, and stored at −80°C.

### 2.3. Measurement of Collagen Level Using Enzyme-Linked Immunosorbent Assay (ELISA)

Collagen type I (COL I) and collagen type III (COL III) in the serum of rat abdominal aorta were determined using collagen type I (COL I) and collagen type III (COL III) ELISA kits (Bio-Swamp Co., Ltd., batch number: RA20416, RA20458).

### 2.4. Determination of p-AKT and CTGF Protein Expression Level Using Western Blot

The middle lobes of lung tissues were lysed in a radioimmunoprecipitation assay lysis buffer (catalog number: BYL40944, Jrdun Biotechnology Co., Ltd., Shanghai, China) for 30 min. Subsequently, the lysate was transferred to Eppendorf tubes and then centrifuged at 12,000 rpm for 30 min at 4°C. The supernatant was transferred to a fresh tube and mixed with an equal volume of 2 *∗* SDS and boiled for 20 min. Then, one sample containing 15 *μ*l of protein was fractionated by PAGE and transferred onto a polyvinylidene fluoride membrane for 60 min. Following blocking nonspecific binding sites with 5% skimmed milk in 1.0 M Tris buffer containing 0.1% Tween-20 for 1 h, membranes were probed with CTGF monoclonal antibody (dilution: 1 : 1000, catalog number: Ab6992, Abcam) and p-AKT monoclonal antibody (dilution: 1 : 500, catalog number: Ab38449, Abcam) at 4°C overnight, washed 3 times with TBST, and incubated with biotin-conjugated secondary antibody (dilution: 1 : 1000, catalog number: A0208, Beyotime Biotechnology, Shanghai, China) for 1 h at 37°C. Mix ECL luminous liquids A and B evenly, followed by clipping in the positive membrane, and then put them in a dark room to avoid light for 5 min. Discard the color liquid and then draw it carefully, and then cover it with a layer of transparent paper. Finally, put it into the imaging system for scanning. The protein expression level of CTGF and p-AKT was quantified by image densitometry, and the ratios of CTGF/GAPDH and p-AKT/GAPDH were statistically analyzed.

### 2.5. Determination of CTGF and AKT mRNA Expression Level Using RT-PCR

Total RNA was isolated from the lung tissues using TRIzol reagent (catalog number: 1596-026, Invitrogen) according to the manufacturer's instructions. RNA was reverse-transcribed using the First Strand cDNA Synthesis Kit (catalog number: #K1622, Fermentas). Quantitative PCR (qPCR) was performed using the SYBR Green RT-PCR Kit (catalog number: #K0223, Thermo Fisher Scientific, Inc., Shanghai, China). The initial denaturation step was at 95°C for 10 min, and then PCR amplification was achieved by 40 cycles at 95°C for 15 sec and at 60°C for 45 sec. The process of RT-PCR was implemented by ABI Prism 7300 (Applied Biosystems, Foster City, CA, USA). Primers used for amplification of the respective gene used in this study are as follows:

  AKT (5′-TGTGGCTGATGGACTCAAACG-3′ and 5′-ATGGCATAGTAGCGACCTGTG-3′)
  CTGF (5′-TGATAGCCTCAAACTCCAAAC-3′ and 5′-ATCCATTGCTTTACCGTCTAC-3′)
  GAPDH (5′-GTCGGTGTGAACGGATTTG-3′ and 5′-TCCCATTCTCAGCCTTGAC-3′)

The level of gene expression was quantified using a standard curve and the comparative CT method normalized to GADPH mRNA expression. We used the formula 2^−ΔΔCT^ to calculate the relative expression levels of genes.

### 2.6. Statistical Analysis

Data were presented as mean ± standard deviation from three separate experiments performed in duplicate. Statistical analysis was performed using SPSS 21.0 software (SPSS, Chicago, IL). One-way analysis of variance was used to compare groups means. Differences were considered significant if *P* < 0.05.

## 3. Results

### 3.1. General Situation of Rats

In the blank control group, rats were active and responsive and had good diet and spirit; their respiratory rate was within normal limits; their fur was smooth and bright, and their weights were increasing. However, in the model control group, after 2-3 days of modeling, both activity and food intake of rats were reduced, respiratory rate became faster than before, and body weight decreased. After 6-7 days of modeling, their fur became dark and gloomy, and they started having arched backs. After 14 days of modeling, they had cyanosis in the outer edges of their limbs and lips, as well as cough and shortness of breath symptoms. After 28 days of modeling, the cyanosis and breath symptoms became worse, and the weight of all rats significantly declined. Additionally, the situation of the rats in the positive medicine control group (methylprednisolone) and the high- and moderate-dose Buyang Huanwu decoction groups was better than that in the model control group on the 7th, 14th, and 28th day, while the difference of the situation of rats between the low-dose Buyang Huanwu decoction group and model control group was insignificant.

### 3.2. Observation of All Rats' Lung Morphology

*Blank Control Group*. There was no abnormal appearance of the lungs, the outline of the lungs was clear, the color of the lungs was pink, and the surface of the lungs was smooth and elastic, with no nodules and bleeding points.

*Model Control Group*. Punctate hemorrhage and focal ecchymosis appeared on the lung surface, the lung volume increased, and the color became dark red on the 7th day. On the 14th day, the lungs became gray, and the surface of the lungs was marked by varying white nodules, with scattered hemorrhages at the edges and smaller volumes in some lung lobes. On the 28th day, the lungs were pale, the surface of the lungs was uneven, there were different sizes of gray nodules and cords like concave sulcus on the lungs, the whole lung volume decreased, elasticity declined, and hardness increased.

**Figure 1:**
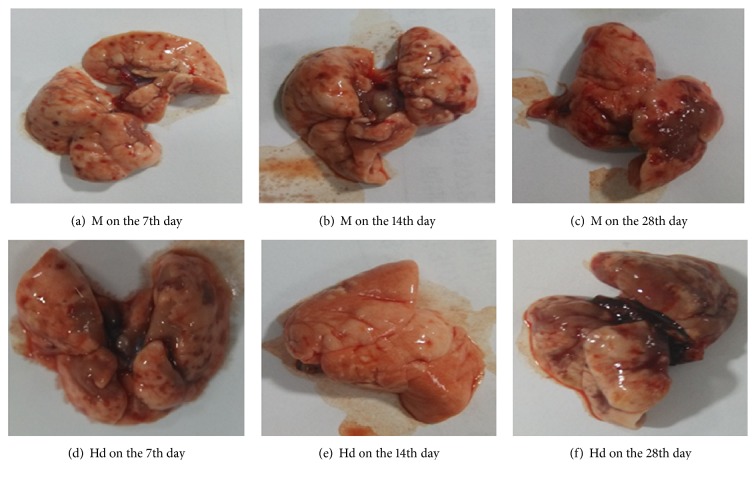
M: the model control group that has been induced into pulmonary fibrosis and treated by 0.9% normal saline. Hd: the high-dose Buyang Huanwu decoction group that is treated by high-dose Buyang Huanwu decoction.

*The Positive Medicine Control Group (Methylprednisolone) and Buyang Huanwu Decoction Groups*. Compared with the model control group, the drug intervention group rats were better than the model control group rats in lung volume, hardness, and elasticity. The lungs of the positive medicine control group (methylprednisolone) and Buyang Huanwu decoction group rats were slightly dark, and the surface of the lungs was slightly rough, as shown in Figures 1(a)–1(f).

### 3.3. The Expression Level of Collagen in Rat Abdominal Aorta

**Figure 2:**
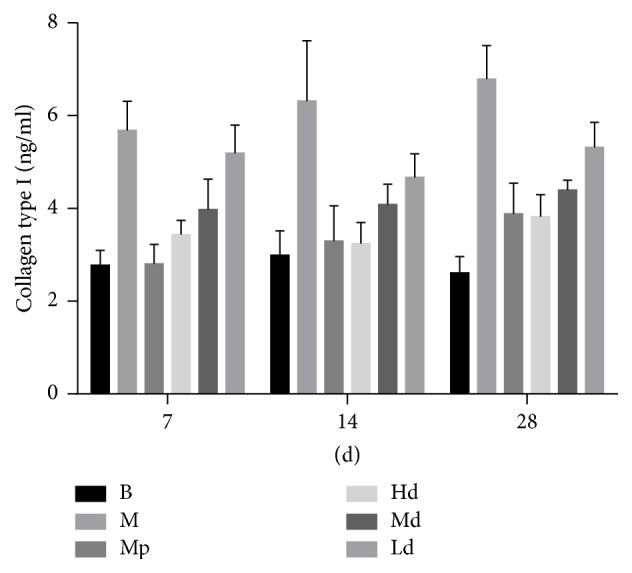
The level of collagen type I. B: the blank control group that is not induced into pulmonary fibrosis. M: the model control group that has been induced into pulmonary fibrosis and treated by 0.9% normal saline. Mp: the positive medicine control group that is treated by methylprednisolone. Hd: the high-dose Buyang Huanwu decoction group that is treated by high-dose Buyang Huanwu decoction. Md: the moderate-dose Buyang Huanwu decoction group that is treated by moderate-dose Buyang Huanwu decoction. Ld: the low-dose Buyang Huanwu decoction group that is treated by low-dose Buyang Huanwu decoction.

**Figure 3:**
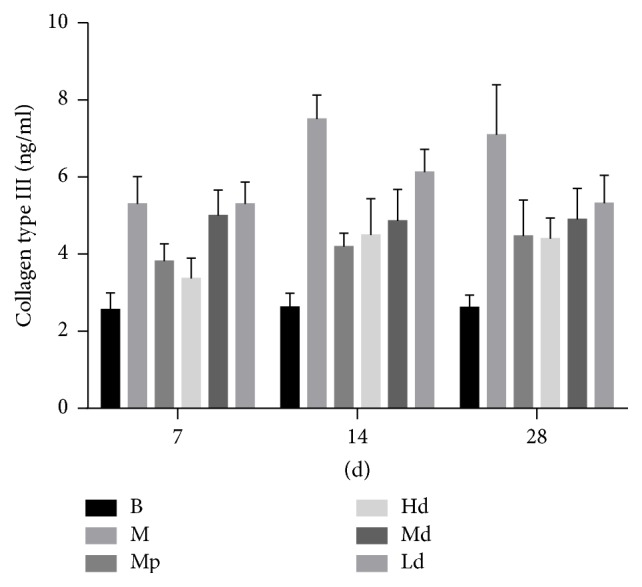
The level of collagen type III. B: the blank control group that is not induced into pulmonary fibrosis. M: the model control group that has been induced into pulmonary fibrosis and treated by 0.9% normal saline. Mp: the positive medicine control group that is treated by methylprednisolone. Hd: the high-dose Buyang Huanwu decoction group that is treated by high-dose Buyang Huanwu decoction. Md: the moderate-dose Buyang Huanwu decoction group that is treated by moderate-dose Buyang Huanwu decoction. Ld: the low-dose Buyang Huanwu decoction group that is treated by low-dose Buyang Huanwu decoction.

To evaluate the variation of cytokines in different agent treatments, expression levels of collagen in the serum of rat abdominal aorta were determined by ELISA. The serum collagen type I level in high- and moderate-dose Buyang Huanwu decoction group rats was significantly lower than that in the model control group on the 7th, 14th, and 28th day (*P* < 0.01). The serum collagen type III level in high-dose Buyang Huanwu decoction group rats was also significantly lower than that in the model control group on the 7th, 14th, and 28th day (*P* < 0.01). Furthermore, the expression level of collagen types I and III in moderate- and low-dose Buyang Huanwu decoction group rats was significantly lower than that in the model control group on the 14th and 28th day (*P* < 0.01) (see Table 1 and Figures 2 and 3).

### 3.4. The Protein Expression Level of CTGF and p-AKT

The ratio to GAPDH was treated as the relative expression level of CTGF and p-AKT in lung tissues. According to the results of western blot, the protein expression level of p-AKT in the high-dose Buyang Huanwu decoction group rats was significantly lower than that in the model control group on the 7th, 14th, and 28th day (*P* < 0.01). The protein expression level of p-AKT in the moderate-dose Buyang Huanwu decoction group rats was significantly lower than that in the model control group on the 7th and 14th day (*P* < 0.01). The protein expression level of p-AKT in the low-dose Buyang Huanwu decoction group rats was only significantly lower than that in the model control group on the 7th day (*P* < 0.05).

**Figure 4:**
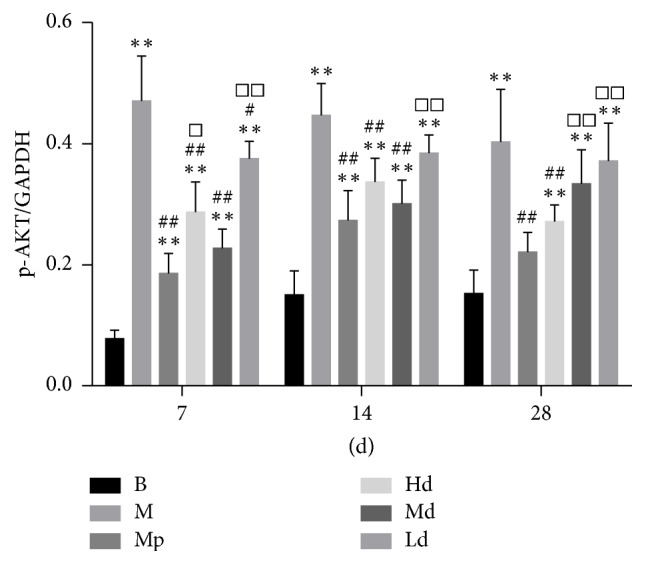
The expression level of p-AKT. Determination of p-AKT and CTGF protein expression level by western blot. Compared with the B group (^*∗∗*^*P* < 0.01); compared with the M group (^#^*P* < 0.05, ^##^*P* < 0.01); compared with the Mp group (^□^*P* < 0.05, ^□□^*P* < 0.01). B: the blank control group that is not induced into pulmonary fibrosis. M: the model control group that has been induced into pulmonary fibrosis and treated by 0.9% normal saline. Mp: the positive medicine control group that is treated by methylprednisolone. Hd: the high-dose Buyang Huanwu decoction group that is treated by high-dose Buyang Huanwu decoction. Md: the moderate-dose Buyang Huanwu decoction group that is treated by moderate-dose Buyang Huanwu decoction. Ld: the low-dose Buyang Huanwu decoction group that is treated by low-dose Buyang Huanwu decoction.

**Figure 5:**
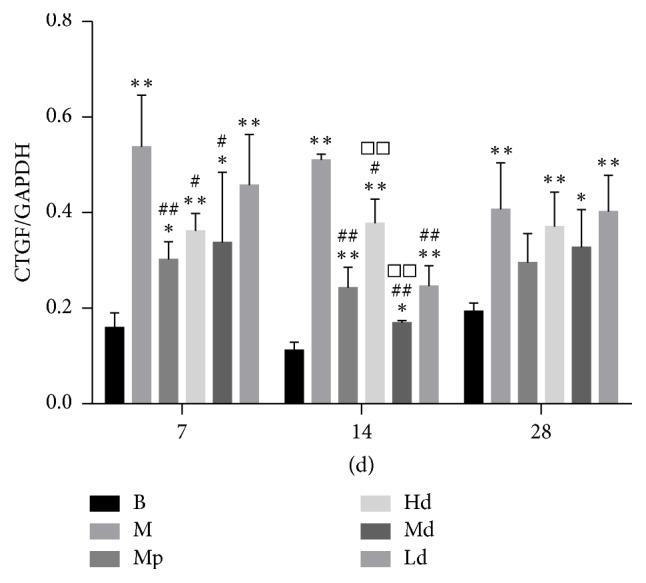
The expression level of CTGF. Determination of p-AKT and CTGF protein expression level by western blot. Compared with the B group (^*∗*^*P* < 0.05, ^*∗∗*^*P* < 0.01); compared with the M group (^#^*P* < 0.05, ^##^*P* < 0.01); compared with the Mp group (^□□^*P* < 0.01). B: the blank control group that is not induced into pulmonary fibrosis. M: the model control group that has been induced into pulmonary fibrosis and treated by 0.9% normal saline. Mp: the positive medicine control group that is treated by methylprednisolone. Hd: the high-dose Buyang Huanwu decoction group that is treated by high-dose Buyang Huanwu decoction. Md: the moderate-dose Buyang Huanwu decoction group that is treated by moderate-dose Buyang Huanwu decoction. Ld: the low-dose Buyang Huanwu decoction group that is treated by low-dose Buyang Huanwu decoction.

**Figure 6:**
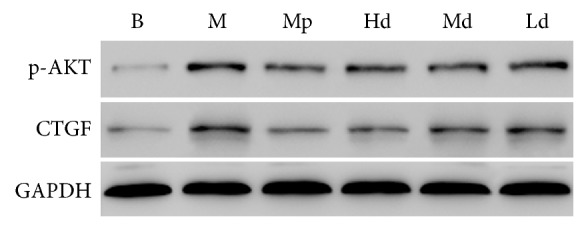
The western blot test results on the 7th day. B: the blank control group that is not induced into pulmonary fibrosis. M: the model control group that has been induced into pulmonary fibrosis and treated by 0.9% normal saline. Mp: the positive medicine control group that is treated by methylprednisolone. Hd: the high-dose Buyang Huanwu decoction group that is treated by high-dose Buyang Huanwu decoction. Md: the moderate-dose Buyang Huanwu decoction group that is treated by moderate-dose Buyang Huanwu decoction. Ld: the low-dose Buyang Huanwu decoction group that is treated by low-dose Buyang Huanwu decoction.

**Figure 7:**
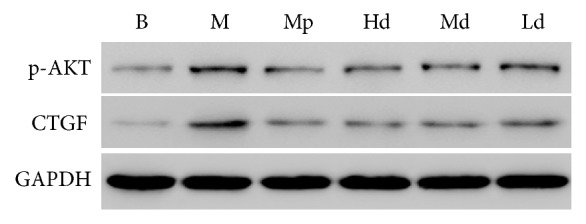
The western blot test results on the 14th day. B: the blank control group that is not induced into pulmonary fibrosis. M: the model control group that has been induced into pulmonary fibrosis and treated by 0.9% normal saline. Mp: the positive medicine control group that is treated by methylprednisolone. Hd: the high-dose Buyang Huanwu decoction group that is treated by high-dose Buyang Huanwu decoction. Md: the moderate-dose Buyang Huanwu decoction group that is treated by moderate-dose Buyang Huanwu decoction. Ld: the low-dose Buyang Huanwu decoction group that is treated by low-dose Buyang Huanwu decoction.

**Figure 8:**
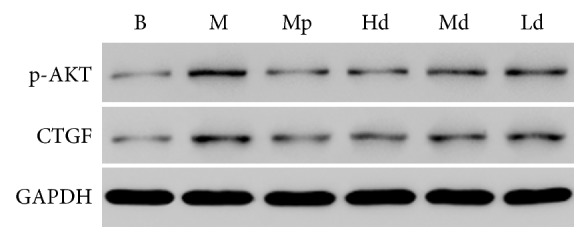
The western blot test results on the 28th day. B: the blank control group that is not induced into pulmonary fibrosis. M: the model control group that has been induced into pulmonary fibrosis and treated by 0.9% normal saline. Mp: the positive medicine control group that is treated by methylprednisolone. Hd: the high-dose Buyang Huanwu decoction group that is treated by high-dose Buyang Huanwu decoction. Md: the moderate-dose Buyang Huanwu decoction group that is treated by moderate-dose Buyang Huanwu decoction. Ld: the low-dose Buyang Huanwu decoction group that is treated by low-dose Buyang Huanwu decoction.

Apart from that, the protein expression level of CTGF in the high- and moderate-dose Buyang Huanwu decoction group rats was significantly lower than that in the model control group (*P* < 0.01, *P* < 0.05) on the 7th and 14th day. On the 14th day, the protein expression level of CTGF in the low-dose Buyang Huanwu decoction group rats was also significantly lower than that in the model control group (*P* < 0.01). However, comparing the Buyang Huanwu decoction groups and model control group, there was no significant difference in the protein expression level of CTGF (*P* > 0.05) on the 28th day (see Figures 4–8)

### 3.5. The Gene Expression Level of CTGF mRNA and AKT mRNA

**Figure 9:**
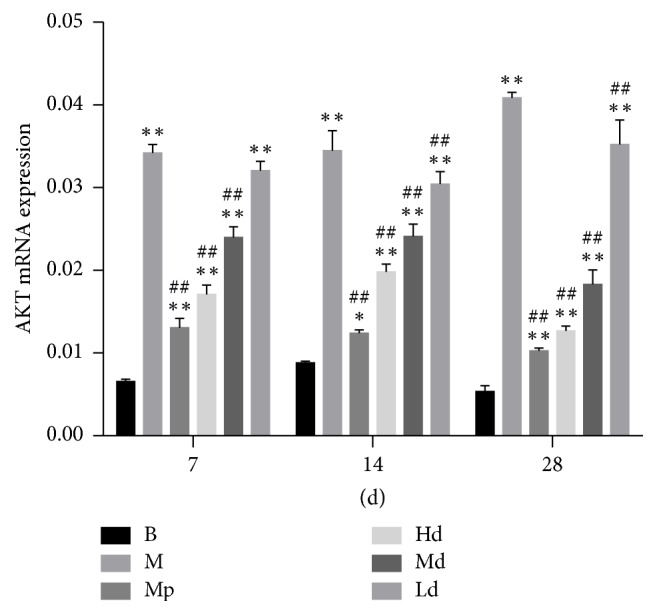
The gene expression level of AKT mRNA. Determination of CTGF and AKT mRNA expression level by RT-PCR. Compared with the B group (^*∗*^*P* < 0.05, ^*∗∗*^*P* < 0.01); compared with the M group (^##^*P* < 0.01). B: the blank control group that is not induced into pulmonary fibrosis. M: the model control group that has been induced into pulmonary fibrosis and treated by 0.9% normal saline. Mp: the positive medicine control group that is treated by methylprednisolone. Hd: the high-dose Buyang Huanwu decoction group that is treated by high-dose Buyang Huanwu decoction. Md: the moderate-dose Buyang Huanwu decoction group that is treated by moderate-dose Buyang Huanwu decoction. Ld: the low-dose Buyang Huanwu decoction group that is treated by low-dose Buyang Huanwu decoction.

**Figure 10:**
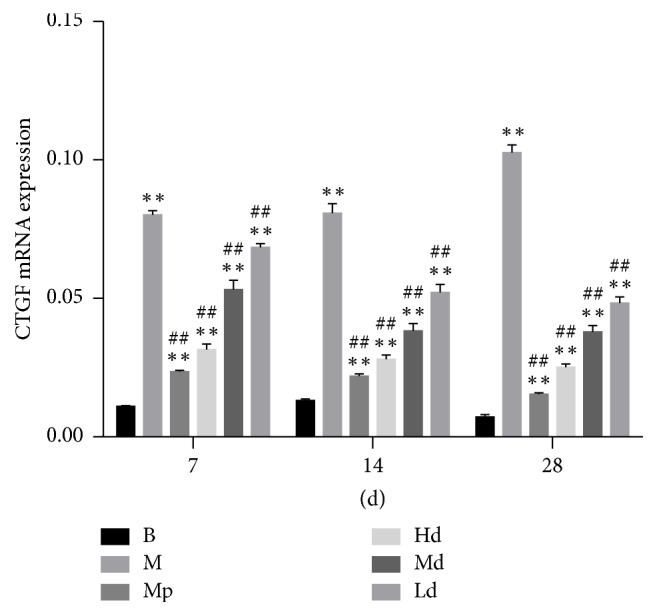
The gene expression level of CTGF mRNA. Determination of CTGF and AKT mRNA expression level by RT-PCR. Compared with the B group (^*∗∗*^*P* < 0.01); compared with the M group (^##^*P* < 0.01). B: the blank control group that is not induced into pulmonary fibrosis. M: the model control group that has been induced into pulmonary fibrosis and treated by 0.9% normal saline. Mp: the positive medicine control group that is treated by methylprednisolone. Hd: the high-dose Buyang Huanwu decoction group that is treated by high-dose Buyang Huanwu decoction. Md: the moderate-dose Buyang Huanwu decoction group that is treated by moderate-dose Buyang Huanwu decoction. Ld: the low-dose Buyang Huanwu decoction group that is treated by low-dose Buyang Huanwu decoction.

According to the real-time PCR test results of CTGF mRNA and AKT mRNA, the gene expression level of CTGF mRNA in the three Buyang Huanwu decoction groups rats and the gene expression level of AKT mRNA in high- and moderate-dose Buyang Huanwu decoction group rats were significantly lower than in the model control group on the 7th, 14th, and 28th day (*P* < 0.01), while the gene expression level of AKT mRNA in the low-dose Buyang Huanwu decoction group rats was only significantly lower than that in the model control group on the 14th and 28th day (*P* < 0.01) (see Figures 9 and 10)

## 4. Discussion

Development of medical research improved the understanding of the pathogenesis of PF and led to the approval of nintedanib and pirfenidone which delay the progress of the disease in some patients with PF. However, the treatment of PF still remains poor, and the clinical efficacy of the treatment of PF needs to be improved. The therapeutic effects of herbs are attributed to unique multitarget and multipathway actions [8]. Buyang Huanwu decoction, as a classical traditional Chinese medicine formula, was widely used for treating circulation system diseases. Buyang Huanwu decoction has the effect of promoting wound healing in chronic skin ulcers of rats by regulating the expression of VEGF, which is a key growth factor for angiogenesis, in granulation tissue of chronic skin ulcers [9]. Perhaps Buyang Huanwu decoction can also treat pulmonary fibrosis by promoting remodeling of the pulmonary vasculature. In this study, we found that, during the lung fibrogenesis induced by BLM in rats, the alveolar inflammation is the most important alteration in the early stage, while in the late stage, the main change is displayed as pulmonary fibrosis [10]. Although the etiology of PF remains unknown, it has been associated with the appearance of fibroblast foci, which are areas rich in activated fibroblasts/myofibroblasts in the background of excessive collagen deposition (mostly type I collagen) indicative of active fibrosis [11, 12]. The results show that Buyang Huanwu decoction can effectively reduce the content of collagen types I and III; this effect is to some extent affected by the dose. CTGF and p-AKT both play an important role in the development of pulmonary fibrosis. In this study, we also found that Buyang Huanwu decoction significantly reduced CTGF and p-AKT protein expression level, and further research found that this effect may be related to the downregulation of these two genes.

The current concept for the development of pulmonary fibrosis, including IPF, is that at least three physiologically balanced processes implicated in the maintenance of lung fibroblasts populations—proliferation, apoptosis of (myo)fibroblasts, and production of ECM—are disturbed [13]. Unlike normal tissue repair, where (myo)fibroblast proliferation is self-limited and cells are eliminated by apoptosis upon completion of repair, in IPF, there is a relentless accumulation of fibroblasts and ECM due to evading apoptosis and sustained cell proliferation [14]. Continuing propagation of myofibroblasts in fibroblastic foci and specific alterations in the ECM structure due to extensive deposition of type I collagen lead to permanently scarred and functionally disabled alveoli [15, 16]. The level of collagen in rat serum can reflect the severity of pulmonary fibrosis. Compared with the M group rats, the high- and moderate-dose Buyang Huanwu decoction significantly reduced the content of serum collagen types I and III at any stage; the moderate-dose Buyang Huanwu decoction significantly reduced the content of serum collagen type I on the 7th, 14th, and 28th day and collagen type III on the 14th and 28th day; the low-dose Buyang Huanwu decoction can also significantly reduce the content of serum collagen types I and III on the 14th and 28th day. The low level of serum collagen types I and III inhibits the extensive deposition of collagen and improves the specific alterations in the ECM structure and functionally disabled alveoli.

Incomplete understanding of the pathogenesis of PF has led to the prevalence of genetic and molecular studies. Downstream mediators of TGF-*β* activity are more appealing, and CTGF, being an immediate early gene induced by TGF-*β*, is being actively investigated [17]. CTGF, like TGF-*β*, increases the synthesis of collagen and fibronectin [18] and regulates developmental, wound healing, and fibrotic responses in numerous tissues including fibroblasts [17, 19–24], endothelial cells [23, 25], and smooth muscle cells [23]. Recently, it has been suggested that key profibrotic proteins such as endothelin-1 (ET-1) and CCN2 [connective tissue growth factor (CTGF)] may operate in tandem with or downstream of TGF-*β* in the fibrotic pathway [26]. In this study, we found a significant decrease of protein expression of CTGF in Hd and Md group rats compared to M group rats on the 7th and 14th day, while on the 28th day, no obvious difference was found between Buyang Huanwu decoction groups and the model control group. This may be associated with the characteristics of pulmonary fibrosis. However, the gene expression level of CTGF mRNA in the three Buyang Huanwu decoction groups rats was significantly lower than that in the model control group rats at any stage. The findings suggest that Buyang Huanwu decoction may alleviate the degree of PF by downregulation of CTGF mRNA.

AKT is a serine/threonine-specific protein kinase that is involved in cellular survival pathways and oncogenesis. It is also a direct target protein downstream of PI3K. Recent studies demonstrated that lung fibroblasts utilize the PTEN-regulated PI3K/AKT-dependent signaling pathway to curb apoptosis and stimulate cell proliferation [27, 28]. p-AKT, which has a main biological function, is the activated state of AKT. To some extent, the expression level of p-AKT that is closely related to the occurrence and development of PF indirectly reflects the activation level of PI3K/AKT signaling pathway. The protein expression level of p-AKT in Hd group rats was significantly lower than that in the M group at any stage. On the 7th and 14th day, the protein expression level of p-AKT in Md group rats was also significantly lower than that in the M group. The gene expression level of AKT mRNA in the three Buyang Huanwu decoction groups was significantly lower than that in the M group at any stage. The effect of Buyang Huanwu decoction in improving pulmonary fibrosis may be related to the downregulation of AKT mRNA and inhibition of the PI3K/AKT signaling pathway.

## 5. Conclusions

Through this study, we can make a conclusion that Buyang Huanwu decoction has a certain therapeutic effect, which may be related to reducing the level of related protein and downregulation of related gene, on the treatment of pulmonary fibrosis, and it can be a therapeutic method for the treatment of pulmonary fibrosis in clinical applications, while the mechanism remains to be explored further at the gene and molecular level.

## Figures and Tables

**Table 1:** The level of collagen in rat serum.

Group	Collagen type I (ng/ml)			Collagen type III (ng/ml)		
	7 d	14 d	28 d	7 d	14 d	28 d
B	2.77 ± 0.32	2.99 ± 0.52	2.61 ± 0.36	2.56 ± 0.43	2.63 ± 0.36	2.62 ± 0.31
M	5.68 ± 0.63^*∗∗*^	6.32 ± 1.30^*∗∗*^	6.79 ± 0.72^*∗∗*^	5.30 ± 0.71^*∗∗*^	7.50 ± 0.63^*∗∗*^	7.10 ± 1.30^*∗∗*^
Mp	2.80 ± 0.42^△△^	3.30 ± 0.76^△△^	3.88 ± 0.66^*∗∗*△△^	3.82 ± 0.45^*∗∗*△△^	4.20 ± 0.35^*∗∗*△△^	4.47 ± 0.93^*∗∗*△△^
Hd	3.43 ± 0.31^△△^	3.24 ± 0.46^△△^	3.82 ± 0.48^*∗∗*△△^	3.37 ± 0.52^*∗*△△^	4.49 ± 0.94^*∗∗*△△^	4.40 ± 0.54^*∗∗*△△^
Md	3.98 ± 0.65^*∗∗*△△□□^	4.08 ± 0.44^*∗*△△^	4.40 ± 0.21^*∗∗*△△^	5.00 ± 0.66^*∗∗*□□^	4.86 ± 0.81^*∗∗*△△^	4.90 ± 0.81^*∗∗*△△^
Ld	5.19 ± 0.61^*∗∗*□□^	4.67 ± 0.51^*∗∗*△△□□^	5.32 ± 0.54^*∗∗*△△□□^	5.30 ± 0.57^*∗∗*□□^	6.13 ± 0.60^*∗∗*△△□□^	5.32 ± 0.73^*∗∗*△△^

Data are presented as mean ± standard deviation. Compared with the B group (^*∗*^*P* < 0.05, ^*∗∗*^*P* < 0.01); compared with the M group (^△△^*P* < 0.01); compared with the Mp group (^□□^*P* < 0.01). B: the blank control group that is not induced into pulmonary fibrosis. M: the model control group that has been induced into pulmonary fibrosis and treated by 0.9% normal saline. Mp: the positive medicine control group that is treated by methylprednisolone. Hd: the high-dose Buyang Huanwu decoction group that is treated by high-dose Buyang Huanwu decoction. Md: the moderate-dose Buyang Huanwu decoction group that is treated by moderate-dose Buyang Huanwu decoction. Ld: the low-dose Buyang Huanwu decoction group that is treated by low-dose Buyang Huanwu decoction.
